# Molecular patterns from a human gut-derived *Lactobacillus* strain suppress pathogenic infiltration of leukocytes into the central nervous system

**DOI:** 10.1186/s12974-020-01959-2

**Published:** 2020-10-06

**Authors:** John Michael S. Sanchez, Daniel J. Doty, Ana Beatriz DePaula-Silva, D. Garrett Brown, Rickesha Bell, Kendra A. Klag, Amanda Truong, Jane E. Libbey, June L. Round, Robert S. Fujinami

**Affiliations:** 1 Department of Pathology, University of Utah School of Medicine, 15 North Medical Drive East, 2600 EEJMRB, Salt Lake City, UT 84112 USA; 2 Department of Oncological Sciences, Huntsman Cancer Institute, 2000 Circle of Hope, 2724 HCI-SOUTH, Salt Lake City, UT 84112 USA

**Keywords:** Lactobacillus, Multiple sclerosis, Experimental autoimmune encephalomyelitis, Gut microbiota, Chemokines, Microbe-associated molecular patterns

## Abstract

**Background:**

Multiple sclerosis (MS) is an inflammatory demyelinating disease that affects 2.5 million people worldwide. Growing evidence suggests that perturbation of the gut microbiota, the dense collection of microorganisms that colonize the gastrointestinal tract, plays a functional role in MS. Indeed, specific gut-resident bacteria are altered in patients with MS compared to healthy individuals, and colonization of gnotobiotic mice with MS-associated microbiota exacerbates preclinical models of MS. However, defining the molecular mechanisms by which gut commensals can remotely affect the neuroinflammatory process remains a critical gap in the field.

**Methods:**

We utilized monophasic experimental autoimmune encephalomyelitis (EAE) in C57BL/6J mice and relapse-remitting EAE in SJL/J mice to test the effects of the products from a human gut-derived commensal strain of *Lactobacillus paracasei* (Lb).

**Results:**

We report that Lb can ameliorate preclinical murine models of MS with both prophylactic and therapeutic administrations. Lb ameliorates disease through a Toll-like receptor 2-dependent mechanism via its microbe-associated molecular patterns that can be detected in the systemic circulation, are sufficient to downregulate chemokine production, and can reduce immune cell infiltration into the central nervous system (CNS). In addition, alterations in the gut microbiota mediated by Lb-associated molecular patterns are sufficient to provide partial protection against neuroinflammatory diseases.

**Conclusions:**

Local Lb modulation of the gut microbiota and the shedding of Lb-associated molecular patterns into the circulation may be important physiological signals to prevent aberrant peripheral immune cell infiltration into the CNS and have relevance to the development of new therapeutic strategies for MS.

## Background

Multiple sclerosis (MS) is an immune-mediated disorder of the central nervous system (CNS) that is characterized by demyelination, inflammation, and axonal damage [1]. While a definitive cause for MS remains elusive, recent attention has turned to the gut microbiota as an environmental factor that may significantly impact diseases. Studies of the fecal microbiome of MS patients have revealed specific bacterial taxa that are reproducibly altered in MS patients compared to healthy controls [2, 3]. Indeed, the transfer of MS-associated microbiota into gnotobiotic mice exacerbates experimental autoimmune encephalomyelitis (EAE), the most common preclinical model of MS, suggesting that the microbiota can play a functional role in diseases [3, 4]. Mechanistically, a growing catalog of microbes and microbial products have been identified that have the ability to skew the neuroinflammatory response away from a proinflammatory phenotype and toward an anti-inflammatory phenotype [5, 6].

Here, we report that a human gut-derived commensal strain of *Lactobacillus* (Lb) is able to suppress the infiltration of leukocytes into the CNS during EAE, independent of skewing the CNS effector T cell response. The protective effect of Lb is dependent on host recognition of Lb-derived microbe-associated molecular patterns (MAMPs) via Toll-like receptor (TLR) 2, as TLR2-deficient mice are not protected by Lb treatment. Furthermore, intestinal microbiota transplants from mice treated with Lb-associated molecular patterns partially ameliorate EAE in recipient mice, suggesting that downstream changes in the gut microbiota also contribute to the effects of Lb. Our findings provide evidence of how gut-derived MAMPs can regulate autoimmunity in the CNS and may support new therapeutic strategies in MS.

## Methods

### Mice

Wild-type C57BL/6J, TLR2-deficient C57BL/6, and wild-type SJL/J mice were obtained from the Jackson Laboratory (Bar Harbor, ME, USA). TLR9-deficient C57BL/6 mice were a kind gift from Dr. Shizuo Akira via Dr. Stella Elkabes. Mice of both sexes were studied. All animal experiments were reviewed and approved by the University of Utah Institutional Animal Care and Use Committee and conducted in accordance with the guidelines prepared by the Committee on Care and Use of Laboratory Animals, Institute of Laboratory Animals Resources, National Research Council. Animals were bred and maintained in the pathogen-free Comparative Medicine Center at the University of Utah on a 12-h light/12-h dark cycle at 22 °C. Germ-free mice were maintained in sterile isolators and verified for germ-free status by plating and PCR of feces. Food and water were available ad libitum. All efforts were made to minimize suffering.

### EAE induction

C57BL/6 mice were injected subcutaneously in the flanks with 200 μL of 1 mM myelin oligodendrocyte glycoprotein (MOG)_35–55_ peptide (Peptide Synthesis Core Facility, University of Utah) emulsified with reconstituted complete Freund’s adjuvant (CFA), composed of incomplete Freund’s adjuvant (Pierce Biotechnology, Rockford, IL, USA) containing *Mycobacterium tuberculosis* H37 Ra (2 mg/mL) (Difco Laboratories, Detroit, MI, USA). Mice were injected intravenously with 100 μL *Bordetella pertussis* (BP) toxin (List Biological Laboratories, Inc., Campbell, CA, USA) at 0.2 μg per mouse on days 0 and 2 following sensitization. MOG_35–55_-induced EAE mice developed a monophasic disease course.

SJL/J mice were injected subcutaneously in the flanks with 200 μL of 1 mM myelin proteolipid protein (PLP)_139–151_ peptide (Peptide Synthesis Core Facility, University of Utah) emulsified with reconstituted CFA, as described above. Mice were intravenously injected with 100 μL BP toxin (List Biological Laboratories, Inc.) at 0.2 μg per mouse on days 0 and 2 following sensitization. PLP_139–151_-induced EAE mice developed a relapsing-remitting (RR) disease course.

### EAE scoring

C57BL/6 mice injected with MOG_35–55_ peptide and SJL/J mice injected with PLP_139–151_ peptide were weighed and scored regularly for clinical signs of disease. Clinical scoring was as follows: 0, no clinical disease; 1, loss of tail tonicity; 2, mild hind leg paralysis with no obvious gait disturbance; 3, mild leg paralysis with gait disturbance; 3.5, unilateral hind limb paralysis; 4, bilateral hind limb paralysis; and 5, moribund or dead. Plots of the clinical score over time include EAE-induced mice that do not develop disease, when applicable. If the mice were paralyzed to the point where they could not feed or groom themselves (moribund), or they lost 20% of their body weight, the mice were euthanized via inhaled anesthetic.

### Flow cytometry

Mice were euthanized through inhaled anesthetic and perfused with phosphate-buffered saline (PBS). Brain and spinal cord tissues were harvested and enzymatically dissociated with collagenase (MilliporeSigma, St. Louis, MO, USA) and DNase I (Roche, Basel, Switzerland) and subsequently mechanically dissociated by vigorous pipetting. Leukocytes were enriched by Percoll (MilliporeSigma) density gradient centrifugation. Cells were treated with Fc blocker (BioLegend, San Diego, CA, USA), stained with the indicated anti-mouse antibodies for 30 min at 4 °C [V500 anti-mouse CD45 (BD Bioscience, San Jose, CA, USA), APC anti-mouse CD11b (eBioscience, San Diego, CA, USA)], and analyzed by flow cytometry. CNS-derived cells were stained and analyzed individually for each mouse. Live cells were determined by forward and side scatter fluorescence on a BD LSRFortessa X-20 Cell Analyzer (BD Bioscience). Flow cytometry data analysis was performed using the FlowJo software (FlowJo, Ashland, OR, USA).

### Histological analysis

Mice were euthanized through inhaled anesthetic and perfused with PBS followed by 4% paraformaldehyde phosphate-buffered solution. For EAE mice, the spinal cords were harvested, divided into 12 transverse portions, embedded in paraffin, and cut into 4-μm-thick tissue sections. To visualize myelin, the sections were stained with Luxol fast blue. Slides were scanned using the Pannoramic MIDI digital slide scanner, visualized using CaseViewer 2.2 digital microscope software, and quantified using the QuantCenter image analysis platform (3D HISTECH Ltd., Budapest, Hungary). The extent of demyelination was calculated in annotated white matter tracts of the spinal cord by subtracting the percent dark blue (myelinated) area of the total white matter area from 100%.

### Adoptive transfer

Congenically labeled CD45.1^+^ leukocytes from the spleen and mesenteric lymph nodes were isolated 6 days post-EAE induction of donors by mechanical dissociation and enriched using ammonium chloride to lyse red blood cells. Leukocytes were suspended in PBS and transferred to CD45.2^+^-recipient mice (10^8^ leukocytes per mouse) by retroorbital injection, with adoptive transfer-naive mice injected retroorbitally with PBS, 1 day prior to EAE induction of recipients.

### Culture of bacteria

*Lactobacillus* sp. was grown aerobically in De Man, Rogosa, and Sharpe (MRS) broth (VWR, Denver, CO, USA) at 37 °C, and identity was confirmed using 16S rRNA-specific PCR followed by sequencing. Heat-killed (HK) Lb was obtained by heating bacteria to 80 °C for 30 min, and killing was confirmed by plating. Colony-forming units (CFUs) of Lb prior to heat-killing was obtained to ensure the equivalent of 10^9^ CFUs were being delivered. In experiments in which live Lb was compared to HK Lb, a bulk preparation of Lb was made, of which a portion underwent the heat-killing procedure. Bulk preparations of Lb were grown overnight, and glycerol stocks were used through the duration of an experiment. Lb-conditioned media were obtained by growing an overnight culture of Lb, collecting the supernatant, and filter sterilizing the solution through a 0.45-μm filter. The absence of viable cells was confirmed by plating.

### Blood-brain barrier (BBB) and blood-spinal cord barrier (BSCB) permeability assay

BBB and BSCB permeabilities were measured by injecting 100 μL of 1% Evans blue dye (in 0.9% saline, 0.22 μm filtered) retroorbitally into mice. The dye was allowed to circulate for 2–4 h before sacrificing the mice. The mice were perfused with PBS for 3 min to clear the dye-carrying blood from the vessels. Brain and spinal cord tissues were weighed and immersed in formamide overnight at 60 °C to elute the dye. The amount of dye per gram tissue was quantified by spectrophotometry.

### Treatment with bacterial products

Mice were treated daily with *Lactobacillus paracasei* ATCC 27092 (Lb), HK Lb, *L. paracasei* ATCC 334, *L. paracasei* ATCC 11582, *L. paracasei* DSM 2649, *L. paracasei* DSM 5622, or vehicle via oral gavage. Mice were gavaged with 10^9^ CFUs or equivalent preparation of HK Lb in 200 μL of PBS.

### Colonization with PBS- and HK Lb-associated microbiota

Conventionally colonized C57BL/6 mice (*n* = 1 donor mouse per group) were treated daily with either PBS or HK Lb for 4 weeks. Donor mice were sacrificed, and the intestinal contents (small intestine to colon) were harvested and resuspended in PBS under anaerobic conditions. Germ-free mice (9–10 weeks old) were colonized with either PBS- or HK Lb-associated microbiota (*n* = at least 10 recipient mice per group) via oral gavage and were induced to develop EAE 6 weeks after colonization.

### TLR2 ligand detection

HEK-Blue mTLR2 cells (InvivoGen, San Diego, CA, USA) were used according to the manufacturer’s instruction to determine the serum concentrations of TLR2 ligands. After passaging according to the manufacturer’s instruction, a flat-bottom 96-well plate was loaded with 25,000 cells per well. Then, 20 μL of PBS, mouse serum, or serial dilutions, starting from 0.1 μg/mL, of Pam3CSK4, a synthetic triacylated lipopeptide, were added to each well. Cells were incubated for 6 h, spun down, and supernatant isolated. Twenty microliters of supernatant was then incubated with 180 μL of QUANTI-Blue solution (InvivoGen) for 15 min. Optical density at 620 nm was then measured using a Biotek Synergy H1M plate reader.

### Serum chemokine analysis

Blood was collected via cardiac puncture, with heparinized syringes, from euthanized mice. The blood samples were centrifuged (6000×*g*, 30 min, 4 °C), and serum was harvested and stored at − 20 °C until use. Serum chemokines (CCL2, CCL3, CCL4, CCL5, CCL11, CCL17, CCL20, CCL22, CXCL1, CXCL5, CXCL9, CXCL10, CXCL13) were assayed using the LEGENDplex Mouse Proinflammatory Chemokine Panel (13-plex) (Biolegend), according to the manufacturer’s directions, and a BD LSRFortessa X-20 Cell Analyzer. LEGENDplex data analysis was performed using the LEGENDplex Data Analysis software (Biolegend).

### Statistical analysis

Statistical analysis was performed using GraphPad Prism 7 (GraphPad Software, San Diego, CA, USA). Statistical significance was determined using one-way ANOVA with Tukey’s multiple comparisons test to compare the multiple groups or Student *t* test to compare the two groups. Log-rank test was used to compare the EAE incidence between the groups. *P* < 0.05 was used to define statistical significance.

## Results

### Live Lb is not necessary to suppress EAE

**Fig. 1:**
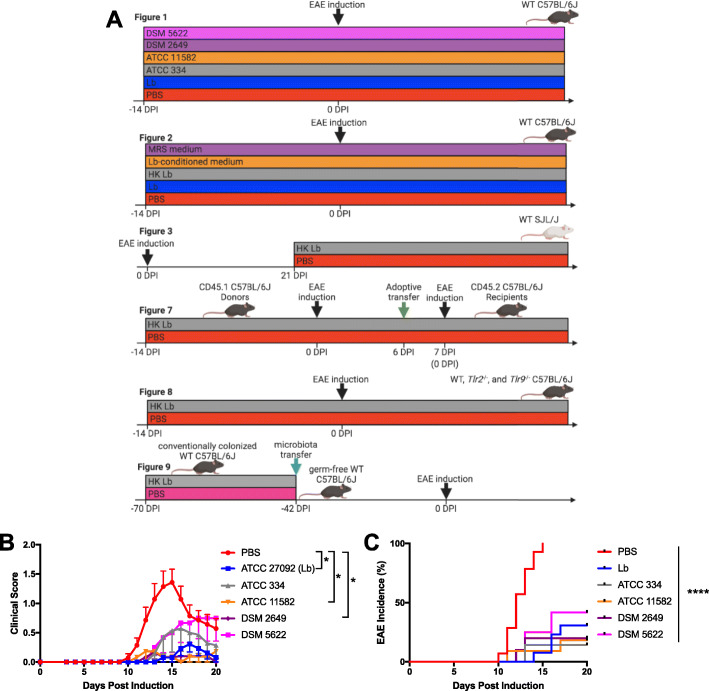
Protective effect in EAE is conserved among *L. paracasei* strains. **a** Overview of in vivo treatment strategies. **b** EAE clinical scores and **c** incidence for C57BL/6J mice orally administered vehicle (PBS) or indicated *L. paracasei* strains daily starting 14 days prior to induction (*n* = at least 10 mice per group). Data are presented as mean ± standard error of the mean (SEM). *P ≤ 0.05, ****P ≤ 0.0001; area under the curve (AUC) one-way ANOVA with Tukey’s multiple comparisons test for **b** and log-rank test for **c**

Previously, through a large-scale screen of EAE-induced mice, we identified *Lactobacillus* sp. as being inversely correlated with disease severity and went on to demonstrate that supplementation of a specific strain of *L. paracasei*, Lb, was sufficient to ameliorate EAE [7]. Given that *Lactobacillus* has been shown to ameliorate EAE in a species- and strain-dependent manner [8], we first asked whether the protective capacity of Lb was conserved among other *L. paracasei* strains. To do this, we treated C57BL/6J mice daily via oral gavage with 10^9^ CFUs with Lb and four other strains of *L. paracasei* isolated from distinct origins (Fig. 1a). We found that the ability to protect against monophasic EAE is largely conserved among different *L. paracasei* strains (Fig. 1b, c). Among the strains that resulted in a significant decrease in disease incidence and severity, Lb was unique in its human gut origin and was used in subsequent experiments.

**Fig. 2:**
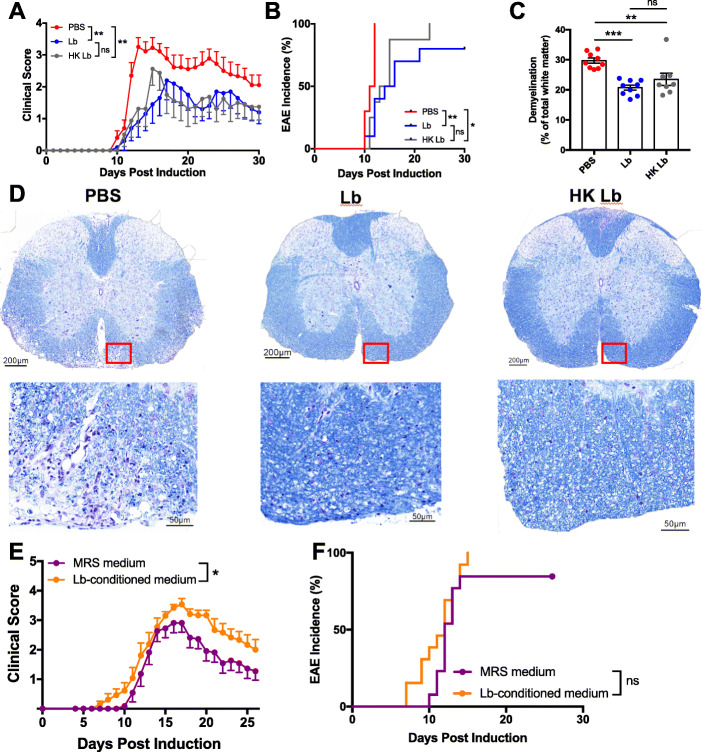
Heat-killed (HK) Lb is sufficient to ameliorate EAE. **a** EAE clinical scores and **b** incidence for C57BL/6J mice orally administered vehicle (PBS), HK Lb, or live Lb daily starting 14 days prior to induction (*n* = at least 10 mice per group). **c** Quantification of demyelination by Luxol fast blue (LFB) staining in the spinal cord cross-sections at 30 DPI (*n* = at least 8 mice per group and at least 6 sections per mouse). **d** Representative LFB staining of the thoracic spinal cord sections from treated mice. **e** EAE clinical scores and **f** incidence for C57BL/6J mice orally administered MRS media or Lb-conditioned media starting 14 days prior to induction (*n* = at least 11 mice per group). Data are presented as mean ± standard error of the mean (SEM) and are representative of two independent experiments. *P ≤ 0.05, **P ≤ 0.01, ***P ≤ 0.001. ns, not significant; AUC one-way ANOVA with Tukey’s multiple comparisons test for **a**, log-rank test for **b** and **f**, one-way ANOVA with Tukey’s multiple comparisons test for **c**, and AUC Student *t* test for **e**

In order to investigate the Lb-derived products required to ameliorate EAE, we treated mice daily with either 10^9^ CFUs of live Lb, an equivalent preparation of HK Lb, or PBS via oral gavage starting 2 weeks prior to EAE induction (Fig. 1a). Monitoring disease incidence and severity, we found that, as before, live Lb-treated animals exhibited lower EAE incidence and severity compared to PBS-treated animals (Fig. 2a, b). Surprisingly, HK Lb-treated animals also exhibited lower EAE incidence and severity compared to PBS-treated animals with protection similar to live Lb-treated animals (Fig. 2a, b). Analysis of spinal cord tissue revealed that both live Lb- and HK Lb-treated mice had less demyelination compared to PBS-treated mice with no difference in demyelination between live Lb- and HK Lb-treated mice (Fig. 2c, d). To further probe whether soluble products secreted by viable Lb contributed to the protective mechanism of Lb, we assessed whether cell-free Lb-conditioned media could limit EAE. We found that mice treated with Lb-conditioned media had the same incidence of EAE and had more severe disease compared to mice treated with sterile De Man, Rogosa, and Sharpe (MRS) media (Fig. 2e, f).

**Fig. 3:**
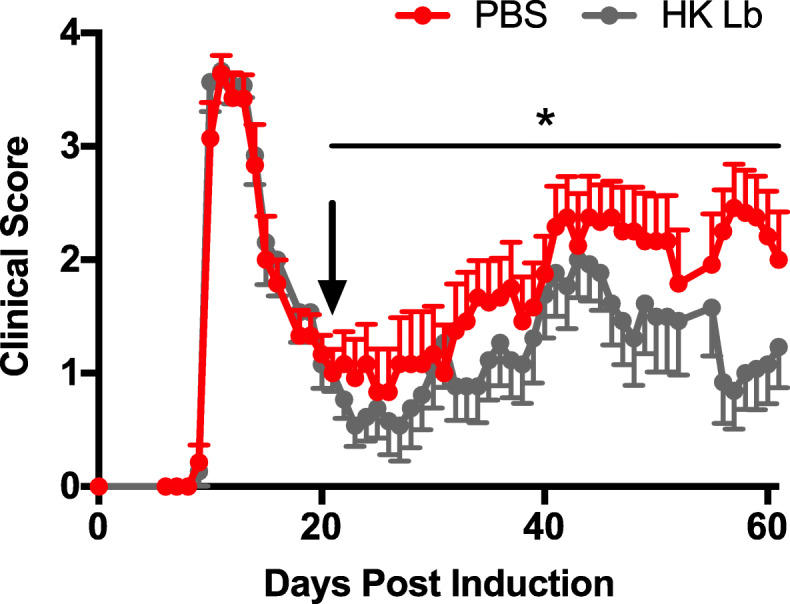
Therapeutic administration of HK Lb reduces the severity of relapse-remitting (RR) EAE. RR-EAE clinical scores for SJL/J mice orally administered PBS or HK Lb daily after the initial attack (indicated by arrow, *n* = at least 12 mice per group). Data are presented as mean ± SEM. *P ≤ 0.05; AUC Student *t* test

We also tested whether HK Lb could be used after the onset of EAE to ameliorate disease (Fig. 1a). We utilized a RR-EAE model in SJL/J mice and initiated treatment with either HK Lb or PBS after the initial attack. We found that therapeutic treatment with HK Lb decreased the severity of the subsequent relapse in RR-EAE (Fig. 3), although the effect of HK Lb was not as pronounced as that observed with prophylactic treatment in monophasic EAE (Fig. 2a). Together, these data demonstrate that the protective effect of Lb is largely conserved among diverse *L. paracasei* strains and that endogenously expressed molecular patterns of Lb are sufficient to prophylactically and therapeutically ameliorate EAE.

### HK Lb limits CNS-infiltrating leukocytes by downregulating circulating chemokines

**Fig. 4:**
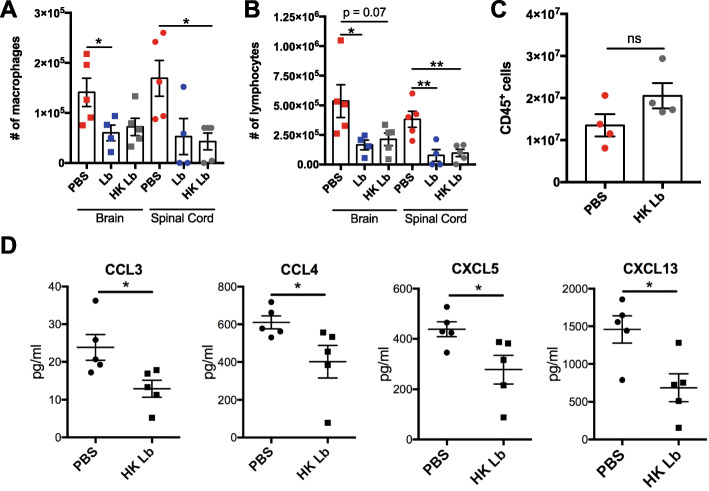
HK Lb reduces CNS-infiltrating leukocytes. **a** The number of CD45^+^CD11b^+^ macrophages in the CNS of EAE-induced mice treated with vehicle (PBS), live Lb, or HK Lb (*n* = at least 4 mice per group) at 14 DPI. **b** The number of CD45^+^CD11b^−^ lymphocytes in the CNS of EAE-induced mice treated with PBS, live Lb, or HK Lb (*n* = at least 4 mice per group) at 14 DPI. **c** The number of CD45^+^ leukocytes in the spleen of EAE-induced mice treated with PBS or HK Lb (*n* = 4 mice per group) at 14 DPI. **d** Concentration of chemokines CCL3, CCL4, CXCL5, and CXCL13 in the plasma of EAE-induced mice treated with PBS or HK Lb (*n* = 5 mice per group) at 14 DPI. Data are presented as mean ± SEM. *P ≤ 0.05, **P ≤ 0.01. ns, not significant; one-way ANOVA with Tukey’s multiple comparisons test for **a** and **b** and Student *t* test for **c** and **d**

**Fig. 5:**
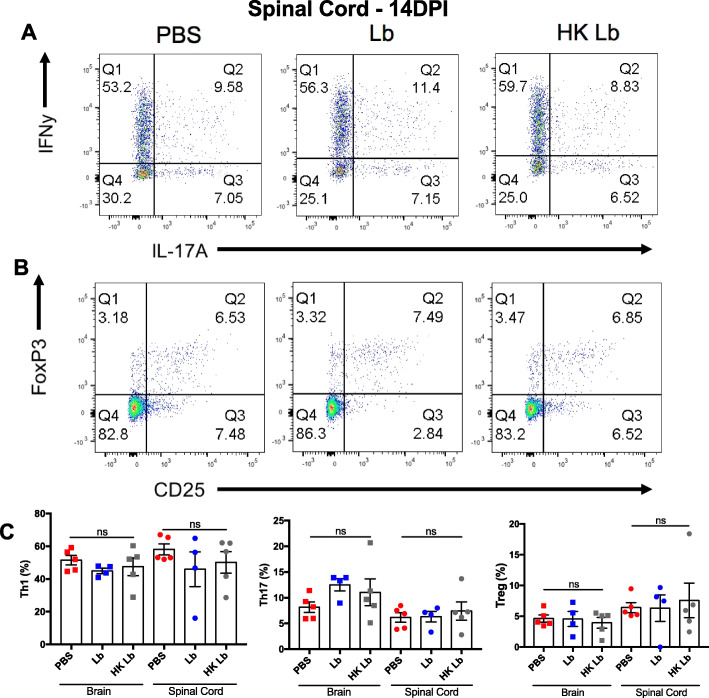
HK Lb does not affect the proportions of CD4^+^IFNγ^+^ T cells, CD4^+^IL-17A^+^ T cells, or Tregs in the spinal cord at peak disease. **a** Representative flow plots of CD4^+^IFNγ^+^ T cells and CD4^+^IL-17A^+^ T cells from the spinal cord of EAE-induced mice treated with PBS, live Lb, or HK Lb at 14 DPI. **b** Representative flow plots of CD4^+^FoxP3^+^CD25^+^ Tregs from the spinal cord of EAE-induced mice treated with PBS, live Lb, or HK Lb at 14 DPI. **c** Proportions of CD4^+^IFNγ^+^ T cells, CD4^+^IL-17A^+^ T cells, and Tregs in the CNS of EAE-induced mice treated with PBS, live Lb, or HK Lb (*n* = at least 4 mice per group) at 14 DPI. Data are presented as mean ± SEM. ns, not significant; one-way ANOVA with Tukey’s multiple comparisons test for **c**

To determine the effect of Lb treatment on monophasic EAE, we isolated leukocytes from the brain and spinal cord of C57BL/6J mice treated with live Lb, HK Lb, and PBS and characterized the CNS infiltrate via flow cytometry. Live Lb- and HK Lb-treated mice had fewer infiltrating macrophages and lymphocytes in both the brain and spinal cord compared to PBS-treated mice (Fig. 4a, b). We also analyzed the proportions of CD4^+^IFNγ^+^ T cells, CD4^+^IL-17A^+^ T cells, and T regulatory cells (Tregs) in the CNS and found no difference between Lb-treated and PBS-treated mice (Fig. 5a–c). Importantly, the number of leukocytes in the spleen between HK Lb-treated mice and PBS-treated mice was not different (Fig. 4c). These results provide evidence that Lb impedes leukocyte migration into the CNS independent of effector T cell modulation.

**Fig. 6:**
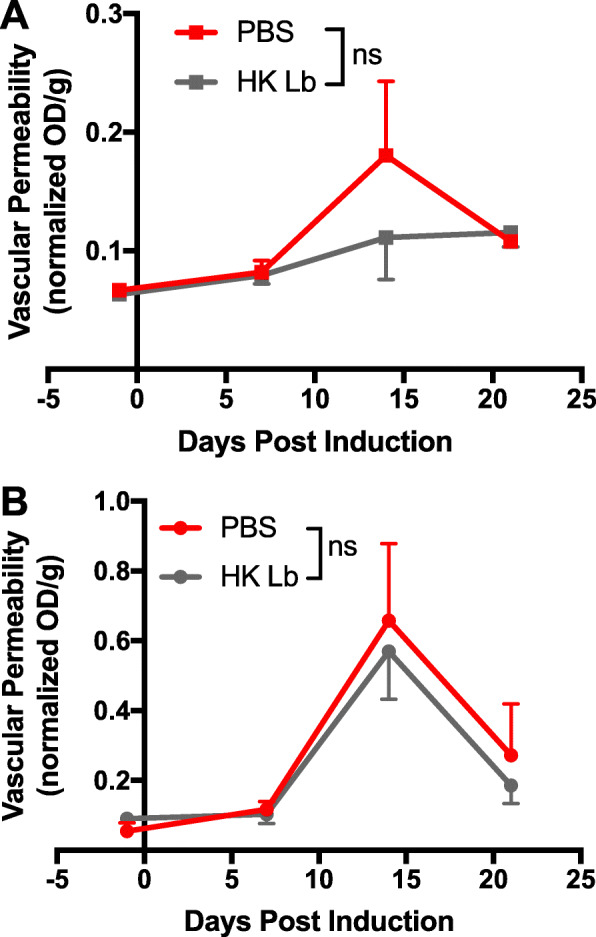
HK Lb treatment does not affect the physical blood-brain nor blood-spinal cord barrier permeability. **a** Evans blue dye permeability into the brain of EAE-induced mice treated with PBS or HK Lb (*n* = at least 3 mice per time point). **b** Evans blue dye permeability into the spinal cord of EAE-induced mice treated with PBS or HK Lb (*n* = at least 3 mice per time point). Data are presented as mean ± SEM. ns, not significant; two-way ANOVA for **a** and **b**

Given the reduction in CNS-infiltrating leukocytes in Lb-treated mice, we obtained the plasma from these mice and measured the level of various chemokines by a multiplex bead-based assay. We observed a reduction in select chemokines CCL3, CCL4, CXCL5, and CXCL13 in HK Lb-treated mice (Fig. 4d). We also tested the permeability of the BBB and BSCB of these mice by Evans blue dye. Mice treated with HK Lb did not differ in BBB and BSCB permeability compared to PBS-treated mice (Fig. 6a, b). Together, these data suggest that Lb decreases the production of chemokines to affect the infiltration of leukocytes into the CNS without affecting the physical permeability of the blood-CNS barrier.

**Fig. 7:**
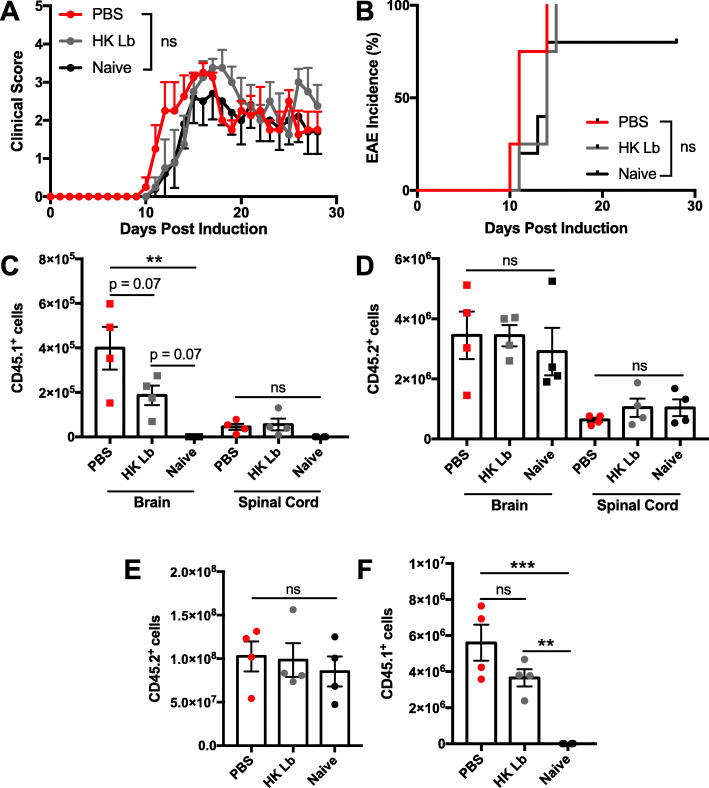
Adoptive transfer of spleen and mesenteric lymph node leukocytes from HK Lb-treated mice does not protect against induced EAE. **a** EAE clinical scores and **b** incidence for adoptive transfer-naive C57BL/6 mice or mice receiving 10^8^ leukocytes from either PBS-treated, EAE-induced donors or HK Lb-treated, EAE-induced donors at − 1 DPI (*n* = at least 4 mice per group). **c** Transferred CD45.1^+^ leukocytes and **d** endogenous CD45.2^+^ leukocytes in the CNS of recipient mice (*n* = 4 per group). **e** Endogenous CD45.2^+^ leukocytes and **f** transferred CD45.1^+^ leukocytes in the spleen of recipient mice (*n* = 4 per group). Data are presented as mean ± SEM. **P ≤ 0.01, ***P ≤ 0.001. ns, not significant; AUC one-way ANOVA Tukey’s multiple comparisons test for **a**, log-rank test for **b**, and one-way ANOVA Tukey’s multiple comparisons test for **c** to **f**

While we did not observe a difference in the proportion of critical helper T cell subsets in the CNS with Lb treatment (Fig. 5a–c), we directly tested whether Lb alters the function of leukocytes to protect against EAE by adoptively transferring leukocytes from the spleen and mesenteric lymph nodes of HK Lb-treated, EAE-induced donor mice into Lb-naive recipient mice 1 day prior to EAE induction. EAE-induced recipient mice that received leukocytes from Lb-treated donors exhibited similar EAE incidence and severity as mice that received leukocytes from PBS-treated donors as well as adoptive transfer-naive mice (Fig. 7a, b). Flow cytometric analysis of CNS leukocytes revealed that donor-derived CD45.1^+^ leukocytes from HK Lb-treated donors were trending to be less prevalent in the brain compared to leukocytes from PBS-treated donors (Fig. 7c). However, there was no difference in the prevalence of donor-derived leukocytes in the spinal cord (Fig. 7c). Importantly, the number of endogenous CD45.2^+^ leukocytes in the CNS (Fig. 7d) and spleen (Fig. 7e) was not different between the groups, and the number of donor-derived CD45.1^+^ leukocytes in the spleen was also not different between the adoptive transfer recipients (Fig. 7f). Thus, Lb treatment likely ameliorates EAE via an immune cell-extrinsic mechanism.

### Host TLR2 signaling is necessary for the protective mechanism of Lb

**Fig. 8:**
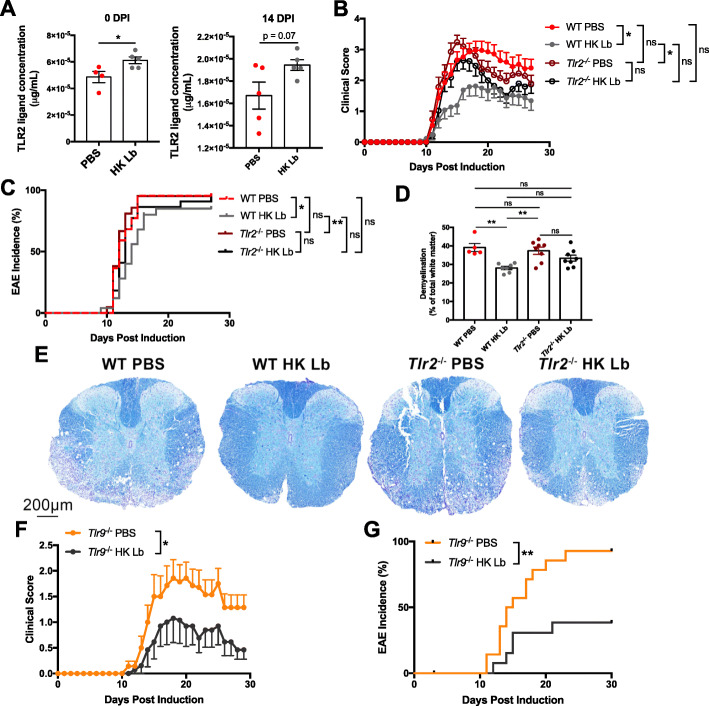
TLR2 is necessary for HK Lb-mediated protection in EAE. **a** Concentration of TLR2 ligand in the plasma of mice treated with PBS or HK Lb at 0 DPI and 14 DPI (*n* = at least 4 mice per group). **b** EAE clinical scores and **c** incidence for wild-type and TLR2-deficient C57BL/6 mice treated with PBS or HK Lb (*n* = at least 21 mice per group). **d** Quantification of demyelination by LFB staining in the spinal cord cross-sections at 30 DPI (*n* = at least 5 mice per group and at least 6 sections per mouse). **e** Representative LFB staining of the thoracic spinal cord sections from treated mice. **f** EAE clinical scores and **g** incidence for TLR9-deficient C57BL/6 mice treated with PBS or HK Lb (*n* = at least 14 mice per group). Data are pooled from two independent experiments and presented as mean ± SEM. *P ≤ 0.05, **P ≤ 0.01. ns, not significant; Student *t* test for **a**, AUC one-way ANOVA with Tukey’s multiple comparisons test for **b**, log-rank test for **c** and **g**, one-way ANOVA with Tukey’s multiple comparisons test for **d**, and AUC Student *t* test for **f**

Since HK Lb is sufficient to suppress EAE, we sought to define the host pattern recognition receptors critical for sensing Lb-associated molecular patterns and leading to a protective phenotype. We hypothesized that TLR2 was a critical signaling node in the protective mechanism of Lb as TLR2 senses peptidoglycan, lipoteichoic acid, and lipoproteins that are major components of the Lb cell wall [9]. Indeed, when we assayed plasma for the presence of TLR2 ligands using TLR2 reporter cells, we found that HK Lb-treated animals had significantly higher concentrations of circulating TLR2 ligand compared to PBS-treated animals at 0 days post-induction (DPI) and a trending increase at 14 DPI (Fig. 8a). To test whether TLR2 was necessary for Lb-mediated regulation of neuroinflammation, we utilized TLR2-deficient mice and compared their response to HK Lb treatment to that of wild-type mice (Fig. 1a). TLR2-deficient mice treated with PBS exhibited similar disease incidence and severity to wild-type mice treated with PBS (Fig. 8b, c). However, TLR2-deficient mice treated with HK Lb did not exhibit significantly less severe disease as observed with wild-type mice receiving HK Lb (Fig. 8b, c). This was also corroborated by histological analysis of demyelination, revealing that while HK Lb-treated wild-type mice exhibited less demyelination than PBS-treated wild-type mice, no such protection was observed with HK Lb-treated TLR2-deficient mice (Fig. 8d, e). To test whether this effect was specific to TLR2, we also measured the response of TLR9-deficient mice to Lb treatment and found that TLR9-deficient mice retained the protective response to Lb treatment (Fig. 8f, g). Thus, TLR2 is specifically necessary for the protective mechanism of Lb.

### HK Lb modulates the gut microbiota to reduce EAE incidence

**Fig. 9:**
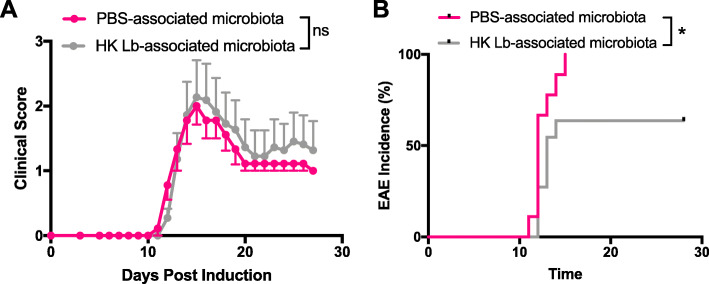
HK Lb reduces EAE incidence by altering the gut microbiota. **a** EAE clinical scores and **b** incidence for germ-free C57BL/6 mice colonized with PBS-associated or HK Lb-associated microbiota (*n* = at least 10 mice per group). Data are presented as mean ± SEM. *P ≤ 0.05. ns, not significant; AUC Student *t* test for **a** and log-rank test for **b**

Finally, we utilized a gnotobiotic approach to probe whether the protective effect of HK Lb was due to direct interaction with the host or an indirect mechanism via HK Lb interaction with other members of the gut microbiota (Fig. 1a). Because germ-free mice exhibit significantly less severe EAE compared to conventionally colonized mice and would be limited in discerning a protective effect of HK Lb directly, we instead compared the development of EAE in germ-free mice colonized with HK Lb-associated gut microbiota or PBS-associated gut microbiota [10]. As before, we treated conventionally colonized C57BL/6 mice daily with either PBS or HK Lb via oral gavage for 4 weeks. We then sacrificed these donor mice and colonized germ-free C57BL/6 mice with their intestinal contents. Monophasic EAE was induced in these mice 6 weeks after the initial colonization. While the overall disease severity was not different between mice colonized with the PBS-associated microbiota or the mice colonized with the HK Lb-associated microbiota (Fig. 9a), mice colonized with the HK Lb-associated microbiota had a significantly lower incidence of EAE (Fig. 9b). Thus, changes in the gut microbiota induced by HK Lb recapitulate some, but not all the features, of HK Lb treatment itself. HK Lb, therefore, likely interacts with both the host and the endogenous gut microbiota to limit CNS autoimmunity.

## Discussion

Given its role in shaping the host immune response, among other physiological processes, significant effort has been devoted to investigating the role of the gut microbiota in MS. *Lactobacillus* sp. has been found to be significantly depleted in MS patients compared to controls [2, 3], mirroring what we and others have observed in EAE [7, 11]. Recently, it has been shown that myelin-specific T helper (Th)17 cells proliferate in the gut prior to the development of neurological symptoms in EAE and are associated with a decrease in gut-derived *Lactobacillus* [11]. Therefore, depletion of *Lactobacillus* may be an early event in MS pathogenesis. Mechanistically, *Lactobacillus* has been proposed to ameliorate EAE by inhibiting pathogenic Th1 and Th17 responses via tryptophan-derived indole metabolites [12–14] and supporting protective Treg responses through an interleukin-10-dependent process [8]. Our work provides an alternative mechanism in which *Lactobacillus*-associated molecular patterns are sufficient to protect against EAE in the absence of *Lactobacillus*-derived metabolites by controlling leukocyte migration to the CNS and modulating the gut-resident microbiota. These are not mutually exclusive pathways as the diverse *Lactobacillus* species that have been shown to ameliorate EAE, including *L. murinis*, *L. reuteri*, *L. paracasei*, *L. helveticus*, and *L. plantarum* strains, likely exert their effects through distinct mechanisms.

In this study, we demonstrate that molecular patterns from Lb, a human gut-derived commensal strain of *L. paracasei*, are sufficient to suppress two different preclinical models of MS (Figs. 2 and 3). Comparing Lb to other *L. paracasei* strains, we show that protection against EAE is conserved among *L. paracasei* strains from diverse sources (Fig. 1). While we do not compare Lb to non-*Lactobacillus* strains, other groups have demonstrated that the administration of bacteria, including *Escherichia coli* MG1655 and segmented filamentous bacteria, can have a neutral or exacerbating effect on EAE [10, 15]. Additionally, Lb-conditioned media exacerbate disease compared to sterile MRS media (Fig. 2e, f). These data argue that the protection we observe in EAE is specifically due to Lb-associated molecular patterns.

Locally, oral administration of HK Lb shifts the gut-resident microbiota to reduce the incidence of EAE (Fig. 9b). It is not clear how Lb-associated molecular patterns alter the gut microbiota, but may involve altering the gut mucosal immune response or the HK Lb itself acting as a substrate for the growth of certain strains. Our gnotobiotic approach demonstrates that products from the microbes associated with HK Lb treatment are sufficient to reduce EAE incidence; however, mice colonized with HK Lb-associated microbiota that develop EAE exhibit more severe disease than mice colonized with PBS-associated microbiota. Combined, this results in no significant effect of HK Lb-associated microbiota on the overall severity of EAE and suggests that repeated HK Lb treatment is necessary for maximal protection (Fig. 9a). The discordant effects of colonization with HK Lb-associated microbiota suggest that HK Lb administration shifts the gut microbiota to prevent disease, but upon disease induction and in the absence of continued HK Lb administration, mice that breakthrough this tolerogenic state adopt more severe disease. Further work is needed to characterize the alterations in the gut microbiota induced by HK Lb and the mechanism by which HK Lb changes the gut microbiota. Still, these data imply that HK Lb both functionally alters the gut microbiota as well as directly interacts with the host as part of its protective mechanism.

Beyond the gut, Lb-associated molecular patterns are also found systemically, attenuating leukocyte infiltration into the CNS (Fig. 4a, b). Interestingly, disease protection conferred by Lb is not associated with modulation of Tregs, CD4^+^IFNγ^+^ T cells, or CD4^+^IL-17A^+^ T cells, which are key to EAE pathogenesis [16], nor is adoptive transfer of Lb-treated leukocytes sufficient to protect against disease (Figs. 5 and 7). This suggests that Lb functions through a leukocyte-extrinsic mechanism and that sustained exposure to Lb-associated molecular patterns is important to ameliorate EAE. Critically, we show that while the physical permeability of the BBB and BSCB is not affected by HK Lb treatment, HK Lb-treated animals exhibit decreased circulating chemokines (Figs. 4d and 6). These chemokines can be produced from non-leukocyte populations including astrocytes and endothelial cells and can facilitate extravasation of peripheral leukocytes into the CNS independent of the physical integrity of CNS barriers [17]. The importance of Lb-associated molecular patterns is further substantiated by the higher concentration of TLR2 ligands we observe in the circulation of HK Lb-treated animals and the requirement of host expression of TLR2 for Lb-mediated protection (Fig. 8). During the course of EAE, gut permeability increases [15], and thus, even EAE-induced, PBS-treated animals likely have a variety of gut-derived MAMPs in circulation. Our work provides proof-of-principal that translocation of Lb-derived MAMPs, in particular, from the gut into systemic circulation can ameliorate CNS autoimmunity.

Why would circulating MAMPs regulate peripheral autoimmunity? One possible explanation is that regular exposure to microbiota-derived MAMPs functions in a rheostat-manner to tolerize the host immune response against inappropriate activation. Locally, gut commensal microbes can favor colonization using molecular patterns to modulate the host mucosal immune response [18], and systemically, MAMPs from the microbiota are constantly shed into the circulation under physiological conditions [19]. While the host has mechanisms to clear circulating MAMPs [20], MAMPs are constitutively present in the circulation and their function is not fully understood. A homeostatic level of circulating MAMPs appears to be critical for the development of the immune system [21], though excess circulating MAMPs lead to systemic inflammation [22]. With relevance to MS, systemic administration of a number of MAMPs, including polysaccharide A derived from *Bacteroides fragilis* and extracellular adherence protein of *Staphylococcus aureus* [23], has been shown to benefit preclinical models of neuroinflammation. Adding to these examples, we find that Lb-associated molecular patterns can impede CNS-infiltrating immune cells and ameliorate EAE. We propose that gut commensal *Lactobacillus* may be a major physiological source of circulating MAMPs as many strains express factors that confer bile and acid resistance, allow for direct adherence to the gut epithelium, and thus would be better positioned to have their MAMPs translocate into circulation [24].

In support of a major role for Lb-associated molecular patterns in conferring resistance against neuroinflammation, we identify TLR2 as a critical host pattern recognition receptor in the protective mechanism of Lb. MS patients exhibit increased expression of TLR2 in peripheral blood mononuclear cells, cerebrospinal fluid cells, and demyelinating lesions [25], and in line with our own preclinical results, MS patients also exhibit decreased microbiota-derived TLR2 ligands in the blood [26]. Additionally, post-mortem analyses of whole brains from MS patients have identified the presence of peptidoglycan in demyelinating lesions [27, 28], evidencing the ability of MAMPs to reach the CNS parenchyma in a neuroinflammatory context. However, investigations into the functional role of TLR2 in MS have produced mixed results. TLR2-deficient mice have been shown to develop either less severe [29] or equivalent [30] EAE to wild-type mice, though this variability may be due to the differences in the microbiota between mouse strains. In fact, TLR2-deficient mice have been reported to display a distinct gut microbiota compared to wild-type mice of the same background, likely due to the differences in the immune response to commensal organisms, with TLR2-deficient mice containing higher levels of *Lactobacillus* than wild-type mice [31]. We strived to minimize variance in the gut microbiota between mouse strains by co-housing mice of different genotypes starting at weaning (4 weeks of age) and continuing through the duration of our experiments to demonstrate the necessity of TLR2.

In apparent opposition to our results, adjuvants used to induce EAE contain TLR ligands, including molecules recognized by TLR2. Additionally, *Staphylococcus aureus*-derived peptidoglycan, which can act as a TLR2 ligand [32], has previously been shown to be a sufficient adjuvant to induce EAE [33]. However, the acute high dose subcutaneous injection of adjuvant is a substantially different context than the chronic low-dose oral administration of HK Lb in this work. While it is possible that these adjuvants may have effects on TLR signaling in our in vivo experiments, this is controlled for in our experimental paradigm as both PBS-treated and Lb-treated mice receive adjuvant. Our data is in agreement with other studies that have demonstrated decreased EAE severity with repeated administration of TLR2 agonists, synthetic or microbiota-derived [34]. These results suggest that while acute TLR2 activation can exacerbate neuroinflammation, prolonged repeated TLR2 signaling may dampen the response to subsequent inflammatory stimuli [34]. Mechanistically, our work demonstrates that daily HK Lb treatment decreases circulating proinflammatory chemokines CCL3, CCL4, CXCL5, and CXCL13 in response to EAE induction (Fig. 4d). Interestingly, these chemokines and/or their respective receptors have been observed to be elevated in MS patients [35], suggesting that Lb-associated molecular patterns result in TLR2-dependent tolerance of a clinically relevant pathway.

## Conclusions

In summary, our findings demonstrate that gut-derived Lb-associated molecular patterns can limit leukocyte migration into the CNS and ameliorate neuroinflammation. While further work is necessary to determine the specific Lb-associated molecular patterns responsible for protection in EAE, beneficial neuroimmunomodulation appears to be a relatively conserved trait among *L. paracasei* strains. Indeed, a pilot study of a probiotic cocktail, VSL3, containing *Lactobacillus* sp. in MS patients has shown beneficial immunomodulatory effects [36]. We postulate that *Lactobacillus* is an important source of protective molecular patterns that are depleted in the context of myelin-specific autoimmunity. HK Lb interacts with other members of the gut microbiota to benefit neuroinflammation. HK Lb supplementation also restores systemic, low-level MAMP signaling; restricts chemokine production; and reduces infiltration of peripheral immune cells into the CNS. As we see prophylactic and therapeutic suppression of demyelinating disease using *Lactobacillus*-associated molecular patterns without the need for live bacteria, HK Lb warrants further investigation as a novel treatment for MS.

## Data Availability

The datasets used and/or analyzed during the current study are available from the corresponding author on reasonable request.

## Abbreviations

ANOVA: Analysis of variance
AUC: Area under the curve
BBB: Blood-brain barrier
BP: *Bordetella pertussis*
BSCB: Blood-spinal cord barrier
CCL2: C-C motif chemokine ligand 2
CCL3: C-C motif chemokine ligand 3
CCL4: C-C motif chemokine ligand 4
CCL5: C-C motif chemokine ligand 5
CCL11: C-C motif chemokine ligand 11
CCL17: C-C motif chemokine ligand 17
CCL20: C-C motif chemokine ligand 20
CCL22: C-C motif chemokine ligand 22
CFA: Complete Freund’s adjuvant
CFU: Colony-forming units
CNS: Central nervous system
CXCL1: C-X-C motif chemokine ligand 1
CXCL5: C-X-C motif chemokine ligand 5
CXCL9: C-X-C motif chemokine ligand 9
CXCL10: C-X-C motif chemokine ligand 10
CXCL13: C-X-C motif chemokine ligand 13
DPI: Days post-immunization
EAE: Experimental autoimmune encephalomyelitis
HK Lb: Heat-killed *Lactobacillus paracasei* ATCC27092
Lb: *Lactobacillus paracasei* ATCC27092
LFB: Luxol fast blue
MAMP: Microbe-associated molecular pattern
MOG: Myelin oligodendrocyte glycoprotein
MRS: De Man, Rogosa, and Sharpe
MS: Multiple sclerosis
PBS: Phosphate-buffered saline
PLP: Proteolipid protein
RR-EAE: Relapse-remitting experimental autoimmune encephalomyelitis
SEM: Standard error of the mean
Th1: T helper type 1
Th17: T helper type 17
TLR: Toll-like receptor
TLR2: Toll-like receptor 2
TLR9: Toll-like receptor 9
Treg: Regulatory T cell
WT: Wild-type
